# Design and Fabrication of High-Temperature-Resistant Poly(4-methyl-1-pentene) Loaded with Tungsten and Boron Carbide Particles Against Neutron and Gamma Rays

**DOI:** 10.3390/polym17172306

**Published:** 2025-08-26

**Authors:** Ming Yu, Fan Luo, Xiaoling Li, Xianglei Chen, Zhirong Guo

**Affiliations:** Wuhan Second Ship Design and Research Institute, Wuhan 430205, China; ym2010hfut@163.com (M.Y.); lflfluo@gmail.com (F.L.); lixl005@163.com (X.L.); chenxianglei027@163.com (X.C.)

**Keywords:** PMP-based composites, neutron and gamma shielding performance, proportion design, coupling modification, mechanical properties, thermal properties

## Abstract

A novel high-temperature-resistant W-B_4_C-poly(4-methyl-1-pentene) (PMP) composite shielding material against neutron and gamma rays was developed and fabricated. Firstly, utilizing the ^235^U-induced fission spectrum as the source term, the compositional ratio of the W-B_4_C-PMP ternary composite was optimized using the genetic algorithm-based GENOCOPIII program coupled with MCNP simulations. Then, the composite was fabricated through coupling agent modification, melt mixing, and hot pressing. Finally, the effects of coupling modification and tungsten content on the thermomechanical properties of the composite were investigated. Results demonstrated that functional groups from the silane coupling agent KH550 were successfully grafted onto the filler surfaces. For composites containing 30 wt% modified B_4_C and 40 wt% modified W in the PMP matrix, the heat deflection temperature (HDT) increased by 18.5% and 19.1%, respectively, compared to their unmodified counterparts. The impact strength also improved by 31.6% and 5.0%, respectively. The variation trend of the composite’s modulus approximately followed the classical Einstein model, while its tensile strength and flexural strength conformed precisely to the model: $\frac{\sigma_{c}}{\sigma_{m}}=0.88V_{f}^{-0.02}$. Thermal analysis indicated that the composites possessed a melting point exceeding 230 °C, and their thermal stability improved with increasing filler content.

## 1. Introduction

Effective neutron and gamma radiation shielding necessitates the synergistic integration of three fundamental mechanisms: (1) thermalization of fast neutrons through elastic/inelastic scattering, (2) capture of moderated thermal neutrons via high-cross-section absorbers, and (3) attenuation of penetrating gamma photons through high-Z materials. These processes dictate that traditional single-component materials cannot achieve the necessary effectiveness, necessitating the use of multifunctional composite materials [1,2,3]. Current composite shielding materials are typically fabricated by embedding functional shielding particles (e.g., boron, tungsten, and gadolinium) within matrix materials such as concrete, metals, or polymers. Specifically, polymer-based shielding composites exhibit distinct advantages, including: (1) exceptional lightweight properties, (2) compact form factor, (3) high concentrations of light elements (particularly hydrogen and carbon), (4) superior processability, and (5) highly tunable material properties. Consequently, these polymer-based composites are extensively employed in weight-critical applications, including small modular reactors (SMRs), mobile nuclear facilities, spacecraft radiation shielding systems, and advanced nuclear personal protective equipment (PPE) [4,5].

However, modern radiation shielding applications require materials that not only provide lightweight and high-efficiency protection but also demonstrate thermal stability at operating temperatures exceeding 200 °C [6]. Conventional polyethylene shielding materials, which exhibit a maximum operational temperature of only 80~100 °C, prove unsuitable for applications requiring thermal stability at 200 °C. Poly(4-methyl-1-pentene) (PMP), sharing the same chemical repeating unit formula as polyethylene, possesses a melting point of approximately 233 °C. This enables it to provide high-temperature resistance without compromising shielding performance, demonstrating significant potential for high-temperature applications [7]. Tungsten, offering advantages such as being environmentally friendly, non-toxic, and enabling volume reduction, serves as an effective lead-free gamma shielding functional particle [8,9]. ^10^B possesses a high thermal neutron absorption cross-section (3837 barns) among nuclei, along with low-energy γ-ray emission upon capture. Unlike lanthanides or precious metals, boron is non-toxic and cost-effective, making it a widely used thermal neutron absorber [10,11].

Therefore, this study aims to develop a novel high-temperature-resistant W-B_4_C-PMP composite shielding material for neutron and gamma-ray protection. The research methodology entails: first, optimizing the composition ratio of the ternary composite using an algorithmic approach; second, based on this optimized ratio, the filler modification using a coupling agent was investigated, followed by fabrication of the ternary composite via melt mixing and hot pressing; Finally, the influence of tungsten content on the composite’s mechanical and thermal properties was systematically investigated.

## 2. Shielding Material Design

In neutron-gamma mixed radiation fields, the W-B_4_C-PMP composite components serve distinct shielding functions: tungsten primarily facilitates fast neutron inelastic scattering and gamma absorption, the PMP matrix exhibits significant neutron scattering at intermediate energies, and boron carbide provides high thermal neutron capture efficiency. This synergistic yet competing functionality constitutes a classic multi-component optimization challenge [12]. A mixed-source term based on the ^235^U-induced fission spectrum [13] was utilized. The composition ratio of the composite shielding material was optimized using the GENECOPIII program [14], which employs a genetic algorithm in conjunction with MCNP simulations. The optimal shielding performance was determined by minimizing the total dose equivalent rate (combined neutron and γ radiation) passing through the plate model.

The objective function of this shielding optimization design is the total equivalent dose rate for neutron and gamma radiation combined:

(1) $$minf\left(X\right)=af_{n}\left(X\right)+bf_{g}\left(X\right)$$

where $f\left(X\right)$ is the target function value, which represents the total equivalent dose rate after the radiation produced by each fission of the source passes through the plate; $f_{n}\left(X\right)$ is the total equivalent dose rate of a single neutron; $f_{g}\left(X\right)$ is the single gamma total equivalent dose rate; a, b is the weighting coefficient of each item; X is a vector composed of the mass fractions of the material components, $X=\left[X_{1},X_{2},X_{3}\right]$, where (i = 1, 2, 3) is the mass fraction of each component in the shielding material.

For multi-component material formulations, the compositions are required to satisfy the following constraints:

(2) $$\sum_{i=1}^{i=N}X_{i}=1$$

(3) $$1/\rho \left(X\right)=\sum_{i=1}^{i=N}X_{i}/\rho_{i},\;\rho_{i}\leq D$$

(4) L≤X≤U

Equation (2) is the constraint of the equation, i.e., the sum of the mass fractions of the components in the shielding material is 1; in Equation (3), $\rho \left(X\right)$ is the density function of PMP-based composite shielding material, $\rho_{i}$ is the density of a certain component, and D is the density range satisfied by the shielding material; Equation (4) is interval constraint, L and U are upper and lower limits of shielding material components respectively. The U-induced fission spectrum serves as a neutron-γ mixed field source term, with each fission event releasing 2.407 neutrons and 7.77 γ rays. Consequently, the weight coefficients in Equation (1) are assigned as a = 2.407 (neutrons) and b = 7.77 (γ rays).

Given the fission spectrum characteristics of 2.407 neutrons and 7.77 gamma photons per fission event, neutron shielding requirements become dominant due to the higher neutron yield. This leads to a constrained PMP mass fraction range of 0.5~0.9 in the compositional design. To simultaneously ensure γ shielding performance and thermal neutron absorption while satisfying material mechanical and thermal requirements, the tungsten mass fraction is constrained to 0.1~0.5, and the boron carbide mass fraction to 0.01~0.1, based on prior design and production experience. In this optimization, the boron-tungsten PMP composite shielding material exhibits a density range of 0.928~1.588 g/cm^3^. Consequently, Equation (4) defines the mass ratios of boron carbide, tungsten, and PMP as follows:

(5) $$\left\{\begin{array}{c} 0.01\leq B_{4}C\leq 0.1;\\ 0.1\leq W\leq 0.5;\\ 0.5\leq PMP\leq 0.9 \end{array}\right.$$

**Figure 1:**
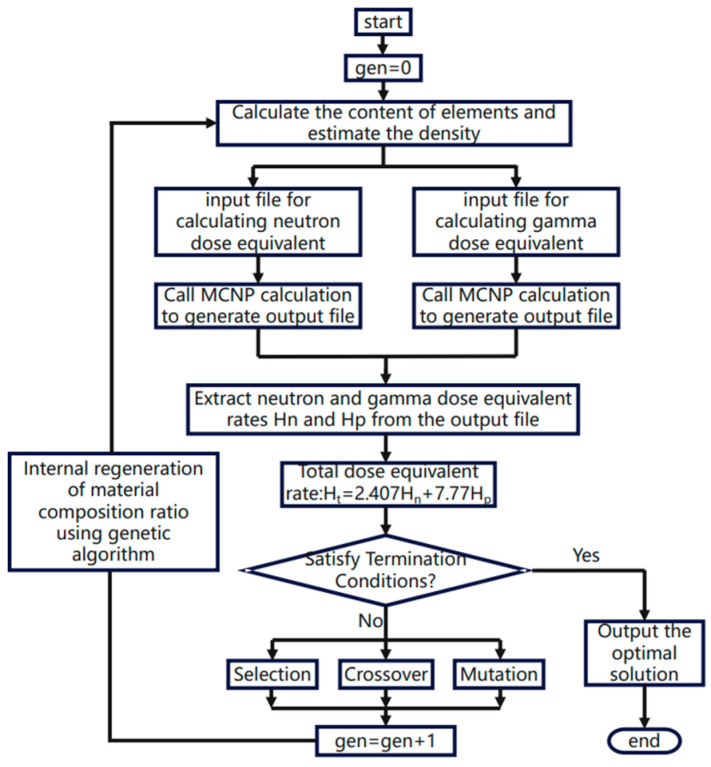
Calculation flow chart of genetic algorithm.

Figure 1 illustrates the computational workflow of the genetic algorithm. The system independently calculates each sub-objective value and feeds back the results to GENOCOP III through a weighted summation of their respective weighting factors. If the design objectives remain unmet, the algorithm repeats the proportion calculation, comparison, and redesign process until convergence to optimal results satisfying all conditions is achieved. This completes the initial distribution ratio design for the composite biological shielding material (Table 1).

To validate the optimized design results, other representative material ratios were selected for comparative calculations. The shielding material ratios used for comparison are listed in Table 2. The MCNP5 program was employed to simulate the attenuation coefficients of Ratios 1–4 for neutrons, γ-rays (including secondary γ-rays), and the total equivalent dose rate from a ^235^U-induced fission source. The attenuation coefficients refer to Formula (6).

**Figure 2:**
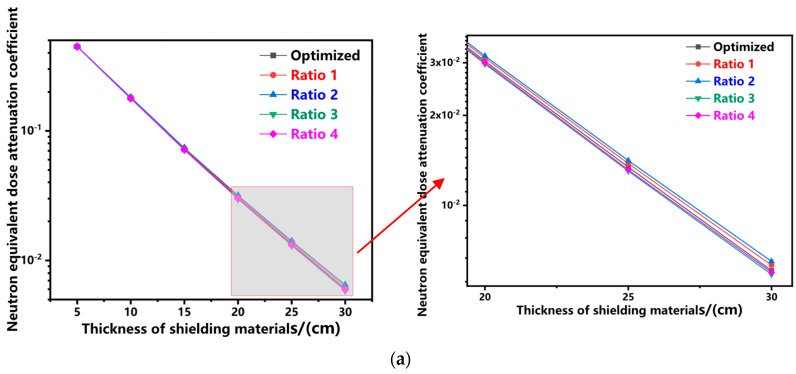

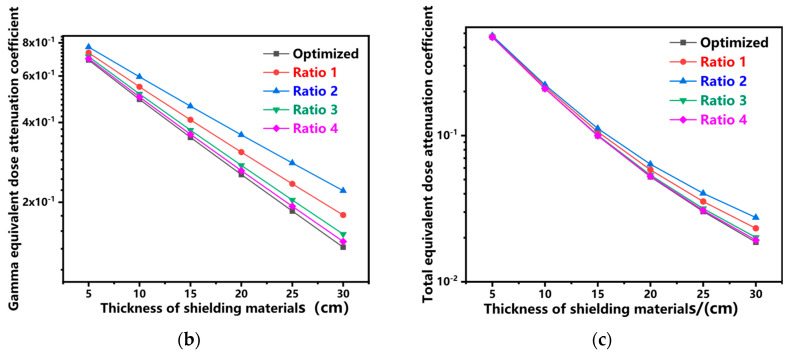
Attenuation Coefficients of Five Materials: (**a**) Neutron equivalent dose attenuation coefficient; (**b**) Gamma equivalent dose attenuation coefficient; (**c**) Total equivalent dose attenuation coefficient.

The equivalent dose rate attenuation coefficient is defined as:

f_i_ = H_i_/H_i0_ (6)

where i = n, r, t represents neutrons, γ-rays, and total equivalent dose rate, respectively; H_i_ is the equivalent dose rate at a point after passing through the shielding material; H_i0_ is the equivalent dose rate at the same point without shielding. The simulation results of attenuation coefficients for the compared shielding materials are shown in Figure 2.

The simulation results in Figure 2 demonstrate that the attenuation coefficients of neutrons, γ-rays, and total equivalent dose rate for all shielding materials follow an exponential decay with increasing thickness, consistent with theoretical attenuation laws, confirming the reliability of calculations. Figure 2a shows a positive correlation between neutron decay ability and hydrogen content. Ratio 3 (highest hydrogen content) shows the strongest neutron decay. Figure 2b shows that the optimization result (with the highest lead content) provides the best gamma ray shielding. Reducing lead content will increase the gamma ray attenuation coefficient (i.e., weaker shielding). Figure 2c indicates that due to its balanced neutron and gamma ray shielding performance, the optimization result has the smallest total equivalent dose attenuation coefficient (optimal overall shielding). In conclusion, comparative simulations with other ratios confirm the correctness and reliability of the optimized design method.

## 3. Materials and Methods

### 3.1. Materials

Raw materials: PMP is procured from Mitsui Chemicals Co., Ltd. (Tokyo, Japan), 10–15 μm tungsten powder is procured from Namako Ruiteng Alloy Materials Co., Ltd. (Xingtai City, China), W1.5 boron carbide is procured from Mudanjiang Hongda Boron Carbide Co., Ltd. (Mudanjiang, China), KH550 (γ-aminopropyl triethoxysilane) is procured from Shanghai Macalline Biochemical Technology Co., Ltd (Shanghai, China).

Surface modification of B_4_C and W powders: The silane coupling agent (KH550) was employed to modify the interfacial interaction of B_4_C and W particles. Firstly, B_4_C and W particles were dispersed in ethanol and then ultrasonicated for 1 h at room temperature. This process is conducive to the formation of hydroxyl groups on the surface of the particles. Then, 2 wt% of KH550 solution was added dropwise into the above suspension, followed by stirring for 5 h at 80 °C to complete the silylation reaction on the particles surface. Finally, the surface-modified particles were collected and dried at 80 °C for 6 h and further ground to obtain fine particles.

Composite material preparation: To investigate the coupling modification effects of powders and the influence of tungsten content on mechanical and thermal properties, the composites were fabricated according to the ratios listed in Table 3. The preparation process involved: (1) mixing PMP, boron carbide powder, and tungsten powder in specified proportions; (2) hot-pressing the blended mixture into test samples. Prior to modification, tungsten powder underwent acid pickling and activation treatments [15]. Two powder types were used: coupling-modified powders (m-B_4_C and m-W) and unmodified raw powders.

### 3.2. Characterization Methods

The surface morphology of the samples was examined using a TESCAN MIRA3 field-emission scanning electron microscope (FE-SEM) (TESCAN, Brno, Czech Republic) operating at 10 kV acceleration voltage. A Bruker INVENIO-R spectrometer (Bruker, Billerica, MA, USA) was employed to collect spectra in the 4000–400 cm^−1^ range at 4 cm^−1^ resolution using the KBr pellet technique for functional group identification. Surface chemical states were analyzed using a Thermo ESCALAB 250Xi (Thermo Fisher Scientific, Waltham, MA, USA) system with monochromated Al Kα radiation.

JJ-20 pendulum impact tester was used to assess dynamic load performance. Tensile tests were conducted on an Instron 5969 universal testing machine at a constant strain rate of 5 mm/min. Using the same equipment, flexural properties were determined by setting a 64 mm span.

Heat deflection temperature (HDT) of the material under 0.45 MPa load was measured using a WKW-300 Vicat/HDT (Instron, Norwood, MA, USA) tester. Thermal stability was monitored via Thermogravimetric Analysis (TGA) using a Q50 instrument (Nissan, Yokohama, Japan) at a heating rate of 10 °C/min under nitrogen atmosphere (flow rate: 50 mL/min). Melting behavior was analyzed using a Q2000 differential scanning calorimeter (DSC) (TA Instruments, New Castle, DE, USA) with aluminum crucibles at a heating rate of 5 °C/min under nitrogen protection.

## 4. Results and Discussion

### 4.1. Powder Coupling Modification

**Figure 3:**
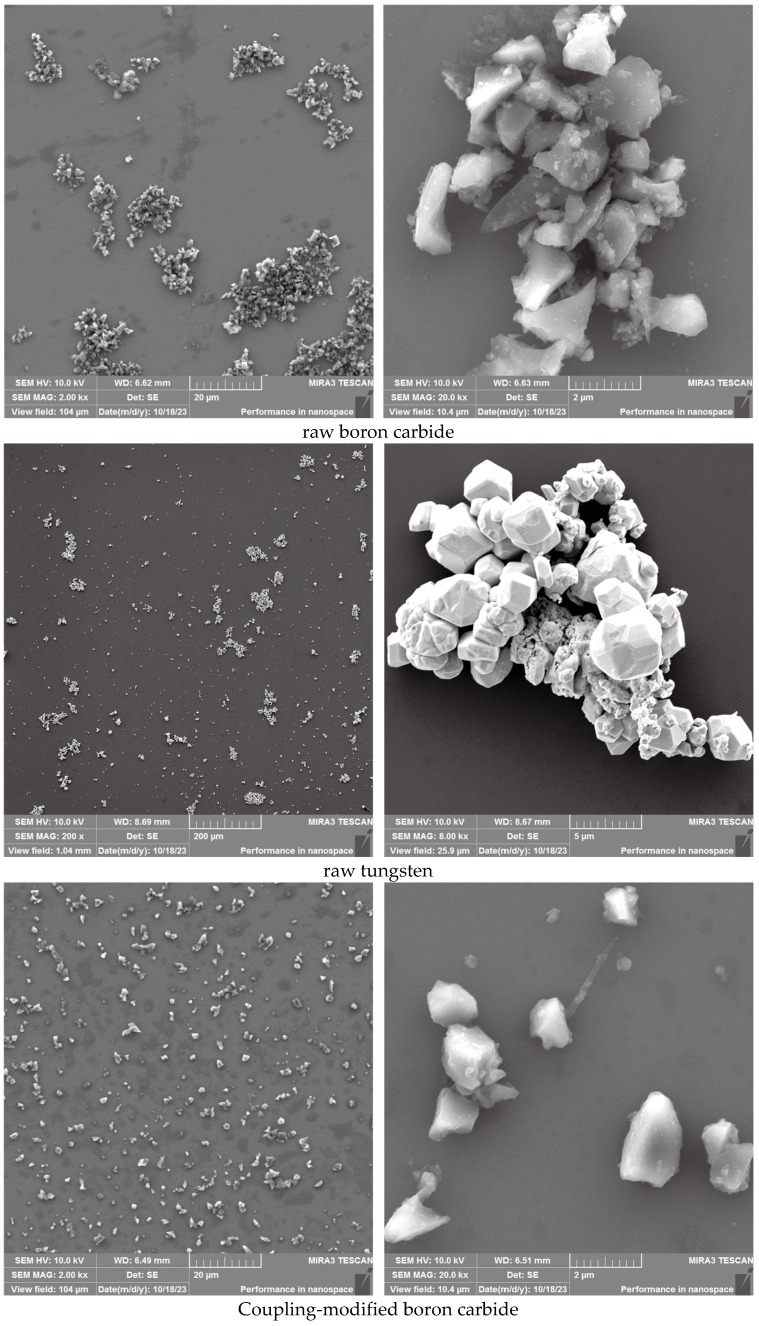

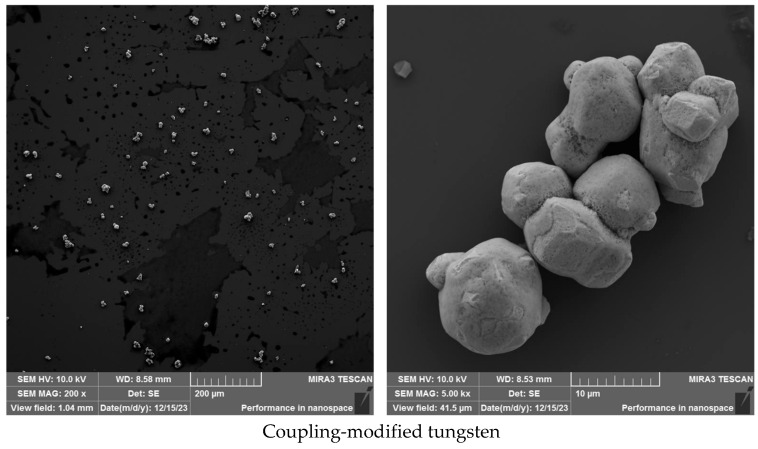
Powder SEM before and after modification.

SEM observations (Figure 3) reveal distinct morphological differences between modified and unmodified powders. The unmodified boron carbide powder exhibits severe agglomeration, whereas the modified counterpart shows significantly reduced particle clustering. Similarly, unmodified tungsten powder demonstrates grape-like agglomerates with sharp edges, whereas KH550-treated samples display markedly improved dispersion with rounded, rough-edged particles. The significant reduction in powder agglomeration achieved through surface modification is attributed to: (1) the permeation and adsorption of the dispersion medium and surface modifier within the powder aggregates, which decreases the Hamaker constant [16] and weakens the attractive forces between powder particles, thereby facilitating effective deaggregation under mechanical force; (2) the adsorption of a layer of surface modifier onto the powder surface, which reduces the free energy of the powder surface and effectively prevents powder agglomeration.

**Figure 4:**
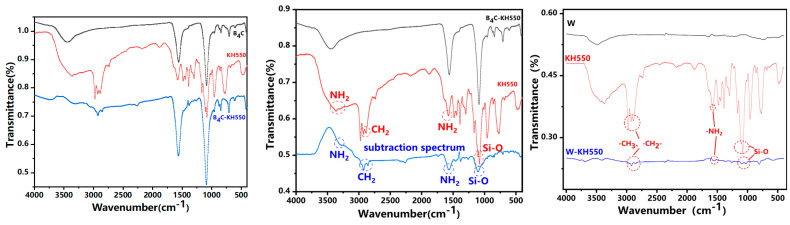
FTIR spectrum of powder before and after modification.

As illustrated in Figure 4, the infrared spectrum of surface-modified B_4_C exhibits two weak absorption peaks at 2924 cm^−1^ (asymmetric stretching) and 2854 cm^−1^ (symmetric stretching), corresponding to the methylene (–CH_2_–) groups [17]. Additionally, the peak at 1562 cm^−1^ overlaps with contributions from both NH_2_ vibrations and B–C bonds, while the peak at 1087 cm^−1^ arises from the Si–O and B–C bond stretching region [18]. These observations confirm the successful grafting of KH550 onto the boron carbide surface. Similarly, new characteristic peaks emerge on the surface of tungsten powder modified with KH550. Specifically, the peaks at 2924 cm^−1^ and 2853 cm^−1^ correspond to the antisymmetric and symmetric stretching vibrations of methylene groups, respectively. The peak at 1574 cm^−1^ represents the absorption of NH_2_, and the peak at 1126 cm^−1^ is characteristic of the Si-O bond. These findings suggest that KH550 has been successfully grafted onto the surface of the modified tungsten powder.

**Figure 5:**
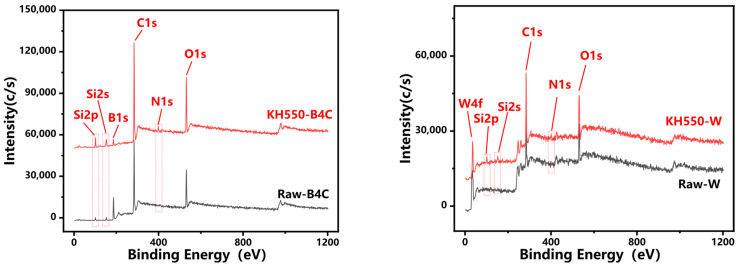
XPS before and after powder modification.

To characterize the surface chemical composition and elemental valence states of boron carbide and tungsten powders before and after modification, X-ray photoelectron spectroscopy (XPS) was performed on both unmodified and modified samples. As shown in Figure 5, the modified powders exhibit two characteristic peaks at 99~104 eV (Si 2p) and 151~155 eV (Si 2s) [19], confirming the presence of silicon originating from the silane coupling agent. Notably, the relative silicon content on modified powder surfaces shows a significant increase compared to unmodified samples, demonstrating successful incorporation of Si atoms from the coupling agent onto both boron carbide and tungsten surfaces. These findings provide conclusive evidence for the effective grafting of silane coupling agent functional groups onto the modified powder surfaces.

Following successful surface modification of the powders, the influence on composite matrix performance was evaluated by fabricating binary composites with both unmodified and modified fillers. For enhanced observational clarity, the composites were formulated with 30% B_4_C-PMP and 40% W-PMP. The prepared samples were subjected to brittle fracture SEM observation, impact test, section observation and load thermal deformation temperature measurement.

**Figure 6:**
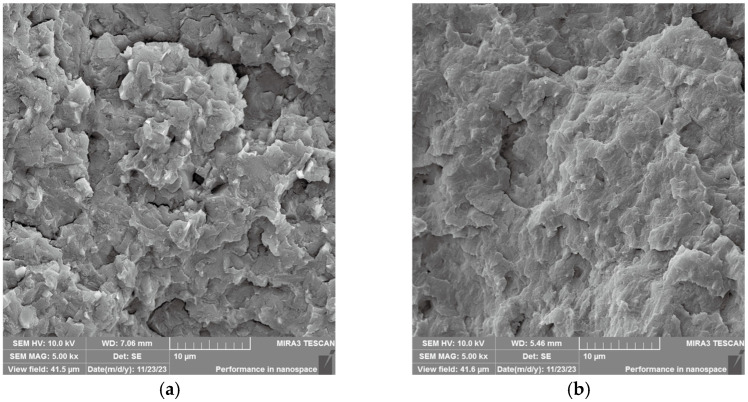

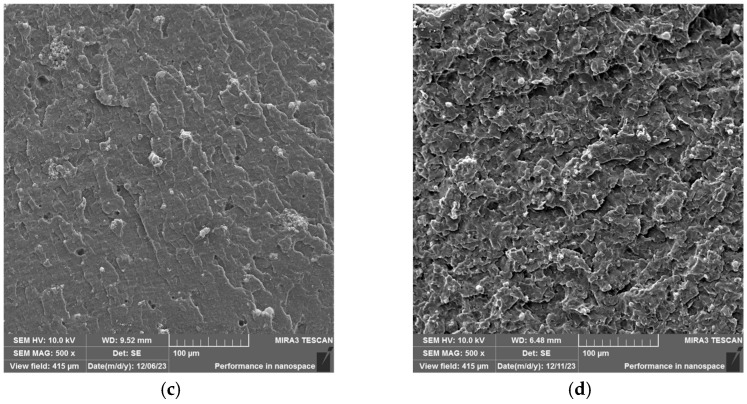
Brittle fracture SEM of binary materials before and after modification: (**a**) raw B_4_C; (**b**) m-B_4_C; (**c**) raw W; (**d**) m-W.

Figure 6a presents the cryofractured SEM morphology of 30% B_4_C-PMP composite with unmodified filler. Notably, distinct interfaces between angular B_4_C particles and PMP matrix are observable. Sharp-edged B_4_C particles remain largely exposed with discernible interfacial gaps and voids (Figure 6a), attributable to poor interfacial adhesion arising from polarity mismatch between inorganic fillers and non-polar polymer matrix [20]. Conversely, Figure 6b demonstrates the modified composite counterpart where silane-treated B_4_C particles exhibit significantly improved matrix wetting. The modified particles appear predominantly encapsulated by resin with minimal exposure, confirming enhanced interfacial bonding [21]. Analogous observations are evidenced in tungsten composites: Unmodified W powder (Figure 6c) displays particle pull-out, smooth fracture planes, and localized agglomeration due to weak interfacial adhesion. In contrast, modified W composites (Figure 6d) reveal reduced agglomeration, roughened fracture surfaces, and effective resin encapsulation of particles.

**Figure 7:**
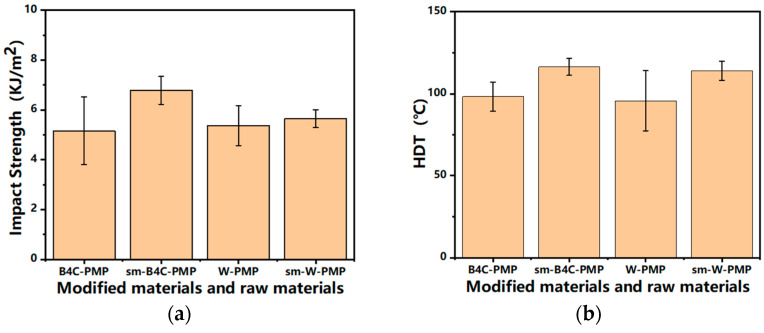
Impact strength and HDT of binary materials before and after modification: (**a**) impact strength; (**b**) HDT.

Figure 7a presents the impact strength results of binary composites (30% B_4_C-PMP and 40% W-PMP) before and after modification, while Figure 7b displays their corresponding heat deflection temperatures (HDT) under load. The coupling-modified powders demonstrate enhanced interfacial adhesion with the matrix, translating to superior impact strength and HDT performance compared to unmodified composites. Notably:30% modified B_4_C-PMP exhibits 18.5% increase in HDT and 31.6% enhancement in impact strength; 40% modified W-PMP achieves 19.1% higher HDT and 5.0% improved impact strength. Post-impact fracture surface analysis further corroborates findings from cryofractured SEM observations, revealing consistent interfacial improvement patterns.

In summary, KH550-modified boron carbide and tungsten powders exhibit enhanced compatibility and interfacial bonding with the organic matrix, leading to superior composite performance. Building upon this established modification protocol, subsequent sections will fabricate composites with varying tungsten contents to systematically examine the effects of tungsten loading on mechanical and thermal properties.

### 4.2. Effect of Tungsten Content on Mechanical Properties

The inorganic particle filled PMP composite material belongs to the “sea-island” structure two-phase system, where the PMP matrix is a continuous phase and the inorganic filler is a dispersed phase. The nature of the filler, the filler content, the interface interaction with the matrix and the aggregation degree in the matrix will obviously affect the reinforcing effect of the filler on the matrix. In the study of particle-filled polymer composites, researchers have proposed a variety of equation models to describe the mechanical properties of composites. The elastic modulus of particle-filled polymer composites is usually determined by the elastic properties of the filled particles and the matrix, the particle content and the length-diameter ratio [22]. When the aspect ratio of the filler particles is 1, i.e., the filler particles are spherical, the modulus of the composite will depend on the modulus of the composition, the content and size of the filler particles. Since the modulus of inorganic particles is usually much higher than that of the polymer matrix, it can be seen from the mixing rule that adding inorganic particles to the matrix can easily increase the modulus of a composite. A number of empirical models have been developed to predict the modulus of particle-filled polymer composites:

Based on the assumption of rigid particles, the Einstein equation for predicting the Young’s modulus of particle composite material [23] is:

(7) ECEm=1+2.5Vf

In Equation (7), $E_{c}$ is the modulus of the composite material, $E_{m}$ is the modulus of the matrix, and $V_{f}$ is the volume fraction of the filler.

Guth [24] introduced particle interaction terms based on Einstein’s equations and proposed a semi empirical equation based on spherical particles:

(8) ECEm=1+2.5Vf+14.1Vf2

**Figure 8:**
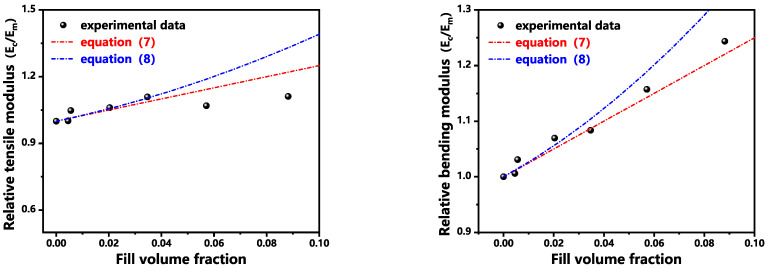
Modulus of Granular-Filled PMP Composites.

The variable definition refers to Formula (7). In the equation, the linear term corresponds to the strengthening effect of individual filler particles, while the quadratic term represents the contribution from particle-particle interactions. Given that the elastic modulus of the filler significantly exceeds that of the matrix material, the incorporation of inorganic particles markedly enhances the composite stiffness, with the elastic modulus increasing progressively alongside filler volume fraction. As clearly demonstrated in Figure 8, the composite modulus closely aligns with the predictions of classical Equations (7) and (8).

The tensile strength of a composite depends on the weakest fracture path throughout the material. The inorganic filler influences the tensile strength in two ways: one is the weakening effect caused by the stress concentration caused by the filler, and the other is the enhancement effect caused by the filler that may hinder the microcrack growth. In some composite materials, the weakening effect dominates, resulting in the tensile strength of the composite material lower than the matrix; In some composite materials, the reinforcing effect dominates, resulting in the strength of the composite material being greater than that of the matrix material.

For filler filled polymer systems with weak interfacial bonds, the Nielsen equation [25] is often used to predict the strength (tensile and bending) of their composites:

(9) $$\sigma_{c}=\sigma_{m}\left(1-V_{f}^{2/3}\right)$$

Based on the assumption that there is no interfacial bonding force between the filler and the matrix, i.e., the load is only borne by the polymer matrix, Nicolais and Nicodemo [26] derived the following expression through simple geometry:

(10) $$\sigma_{c}=\sigma_{m}\left(1-1.21V_{f}^{2/3}\right)$$

In Equations (9) and (10), $\sigma_{c}$ is the composite material strength (tensile and bending), $\sigma_{m}$ is the matrix strength (tensile and bending), and $V_{f}$ is the filler volume fraction.

**Figure 9:**
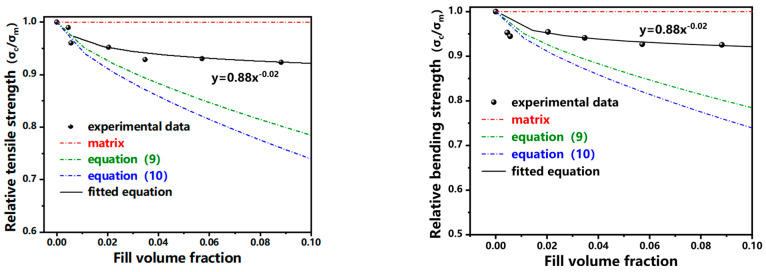
Fitting curve of mechanical property data of filled PMP Composites.

This equation predicts the lower-bound strength of the composite material (strength minimum). When considering perfect interfacial adhesion between filler and matrix, the composite strength can be regarded as equivalent to the matrix strength (upper-bound limit). Consequently, the actual composite strength resides between these theoretical bounds. As demonstrated in Figure 9, experimental tensile and flexural strength data are bracketed by the upper limit (matrix strength) and lower limit (Equation (10)). This confirms that: The coupling-agent-treated inorganic particles achieve partial but effective interfacial bonding with the matrix. Both tensile and flexural strengths meet the equation: $\frac{\sigma_{c}}{\sigma_{m}}=0.88V_{f}^{-0.02}$.

### 4.3. Effect of Tungsten Content on Thermal Properties

In order to study the effect of tungsten content on the thermal properties of composites, thermogravimetric analysis (TGA) and differential scanning calorimetry (DSC) were carried out.

**Figure 10:**
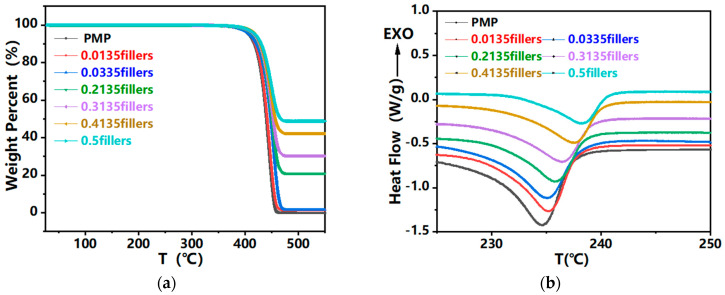
TGA and DSC curves of composites: (**a**) TGA; (**b**) DSC.

Thermal stability of pure PMP and filler filled PMP composites was analyzed using TGA, as shown in Figure 10a, and TGA curves were recorded for the composite at 30 °C to 550 °C. Each curve is divided into three stages: the first stage is volatile matter (water) loss, the weight loss is between 100 °C and 200 °C, and the water loss mainly includes the water evaporated from the composite surface and the water physically absorbed in the material; The second stage is the decomposition of matrix PMP; The third stage is the decomposition of residual carbon. The melting point, Tm (Figure 10b), of both pure PMP and filler filled PMP composites was analyzed using DSC, with melting points greater than 230 °C. Table 4 shows the temperature points corresponding to T_5%_, T_10%_ and T_50%_ in the TGA curve. Obviously, as the tungsten filler content increases, the values of T_5%_, T_10%_ and T_50%_ increase, and the melting point also increases. This is because the packing prevents the thermal movement of the PMP chain segments, resulting in improved thermal stability, which increases with increasing packing content. This is also consistent with the previous HDT rule.

## 5. Conclusions

This study successfully developed a novel high-temperature W-B_4_C-PMP composite for neutron-gamma mixed-field radiation shielding, employing a multi-component optimization strategy. The key findings are systematically summarized below:

(1) The combination of GENOCOPIII program and MCNP program based on genetic algorithm can effectively carry out multi-component optimization design: the optimized mixture ratio of ternary composite material is 0.4865W-0.0135B_4_C-0.5PMP. After comparing multiple sets of material calculations, the accuracy and reliability of the algorithm have been verified. The next step of research should consider the impact of radiation degradation on material properties.

(2) SEM, FTIR and XPS tests before and after powder modification showed that KH550 functional groups have been grafted onto the powder surface. The load thermal deformation temperature of modified 30% B_4_C-PMP and modified 40% W-PMP increased 18.5% and 19.1% respectively, and the impact strength increased 31.6% and 5.0% respectively. Through coupling modification, the compatibility and interface binding force between inorganic powder and organic matrix lead to its enhanced performance.

(3) The modulus of the composite material approximates the classical Einstein equation, and the tensile and bending strengths satisfy the equation: $\frac{\sigma_{c}}{\sigma_{m}}=0.88V_{f}^{-0.02}$.

(4) The melting point of composites is higher than 230 °C, and the thermal stability increases with the increase of filler content. The next step should consider the impact of long-term thermal cycling on the properties of composite materials.

(5) Our comprehensive evaluation of shielding, mechanical, and thermal properties reveals a fundamental trade-off relationship between these performance metrics as functional filler content increases: Shielding efficiency shows an inverse correlation with mechanical strength, and mechanical properties demonstrate competing behavior with thermal stability. To address this multi-dimensional optimization challenge, advanced multi-objective optimization approaches should be implemented to systematically balance these competing performance requirements.

## Figures and Tables

**Table 1:** Optimization design results of composition ratio of boron tungsten PMP composite shielding material (mass ratio).

Component	PMP	W	B_4_C	ρ (g/cm^3^)
Optimal Results	0.500	0.4865	0.0135	1.585

**Table 2:** Composite Material Ratios for Comparison.

No.	Component	PMP	W	B_4_C	Density (g/cm^3^)
1	Optimized	0.5000	0.4865	0.0135	1.585
2	Ratio 1	0.6000	0.3865	0.0135	1.341
3	Ratio 2	0.7000	0.2865	0.0135	1.162
4	Ratio 3	0.5000	0.4000	0.1000	1.514
5	Ratio 4	0.5000	0.4500	0.0500	1.554

**Table 3:** Proportion of PMP-based material preparation.

Content	Component (PMP Base Material)							Notes
	1	2	3	4	5	6	7	
coupling modification	30%B_4_C	30%m-B_4_C	40%W	40%m-W	--	--	--	Binary material
tungsten content	0	1.35%B_4_C	2%W + 1.35%B_4_C	20%W + 1.35%B_4_C	30%W + 1.35%B_4_C	40%W + 1.35%B_4_C	48.65%W + 1.35%B_4_C	Filler modified

**Table 4:** Temperature points corresponding to T_5%_, T_10%_ and T_50%_ in TGA curve.

	PMP	0.0135 Fillers	0.0335 Fillers	0.2135 Fillers	0.3135 Fillers	0.4135 Fillers	0.5 Fillers
T_5%_ (°C)	407.66	408.7	408.87	414.65	416.67	418.85	419.37
T_10%_ (°C)	417.21	418.9	421.75	425.36	427.12	429.14	430.04
T_50%_ (°C)	439.42	441.78	448.07	450.36	452.78	457.45	467.69

## Data Availability

The original contributions presented in this study are included in the article. Further inquiries can be directed to the corresponding authors.
